# Wide-field fluorescent nanodiamond spin measurements toward real-time large-area intracellular thermometry

**DOI:** 10.1038/s41598-021-83285-y

**Published:** 2021-02-19

**Authors:** Yushi Nishimura, Keisuke Oshimi, Yumi Umehara, Yuka Kumon, Kazu Miyaji, Hiroshi Yukawa, Yutaka Shikano, Tsutomu Matsubara, Masazumi Fujiwara, Yoshinobu Baba, Yoshio Teki

**Affiliations:** 1 Department of Chemistry, Graduate School of Science, Osaka City University, Osaka, 558-8585 Japan; 2 Department of Biomolecular Engineering, Graduate School of Engineering, Nagoya University, Nagoya, 464-8603 Japan; 3 Institute of Nano-Life-Systems, Institutes of Innovation for Future Society, Nagoya University, Nagoya, 464-8603 Japan; 4 Quantum Computing Center, Keio University, Yokohama, 223-8522 Japan; 5 Institute for Quantum Studies, Chapman University, Orange , CA 92866 USA; 6 JST PRESTO, Saitama, 332-0012 Japan; 7 Department of Anatomy and Regenerative Biology, Graduate School of Medicine, Osaka City University, Osaka, 545-8585 Japan; 8 Present Address: Institute of Quantum Life Science, National Institutes for Quantum and Radiological Science and Technology, Chiba, 263-8555 Japan

**Keywords:** Nanoscale materials, Microscopy, Microscopy

## Abstract

Measuring optically detected magnetic resonance (ODMR) of diamond nitrogen vacancy centers significantly depends on the photon detectors used. We study camera-based wide-field ODMR measurements to examine the performance in thermometry by comparing the results to those of the confocal-based ODMR detection. We show that the temperature sensitivity of the camera-based measurements can be as high as that of the confocal detection and that possible artifacts of the ODMR shift are produced owing to the complexity of the camera-based measurements. Although measurements from wide-field ODMR of nanodiamonds in living cells can provide temperature precisions consistent with those of confocal detection, the technique requires the integration of rapid ODMR measurement protocols for better precisions. Our results can aid the development of camera-based real-time large-area spin-based thermometry of living cells.

## Introduction

Subcellular thermometry has great potential for studying the molecular mechanisms of temperature-related biological phenomena, such as the variation of cell-death types in photothermal cancer therapy^1–3^, cellular thermotaxis^4–8^, and cellular level thermogenesis^9–13^. Nanodiamond (ND) spin-based thermometry is a novel opto-microwave hybrid technique that can probe subcellular temperatures with distinct photostability^14–18^, various functionalized surfaces^19–21^, and low cytotoxicity^22–26^. The technique is based on optically detected magnetic resonance (ODMR) of electron spins in diamond nitrogen vacancy (NV) color defect centers, where the fluorescence of NDs is measured under microwave irradiation to detect the fluorescence decrease at an electron spin resonance of about 2.87 GHz^27–30^. The ODMR frequency is temperature dependent, and the temperature of NDs can be probed by measuring the ODMR frequency shift^31–42^. Biological applications of this method have been demonstrated for various cultured cells, including fibroblasts^31^, neurons^23^, and stem cells^43^. It has been recently applied to in vivo nematode worms for studying physiological thermogenesis^44^ and the temperature dependence of embryogenesis^45^. These demonstrations have proven the usefulness of ND spin-based thermometry.

To further integrate this technique into biological thermometry, real-time operations and large-area probing are required. ND spin-based thermometry is mainly classified into two types depending on the fluorescence detectors used, that is, photon-counter-based confocal detection and camera-based wide-field detection. Confocal detection is a point-by-point ND probing technique that is widely used for NV-spin manipulation and detection combined with rapid timing control of photon detection and microwave pulsing^27,28^. An example for biological thermometry is the multi-point ODMR method^31,34,36^ that was used for real-time biological thermometry studies^44,45^. Camera-based wide-field detection offers parallel probing of multiple NDs simultaneously that enables spin-based ND thermometry at multiple points in a large field of view^23,46,47^. Combining wide-field ODMR detection with multi-point ODMR methods will enable real-time, large-area monitoring of temperatures under the biological microscope. However, it has been recently reported that multi-point ODMR methods introduce several measurement artifacts in confocal ODMR detection^48^. It is important to analyze the operational process and associated artifacts of wide-field ODMR detection prior to the integration of multi-point ODMR methods, which can add complex artifacts.

This study clarifies factors that can substantially affect temperature measurements in wide-field ODMR detection, including sensitivity degradation from background fluorescence and artifacts from selection of binning regions near target NDs. By properly adjusting these factors, we show that, in living cells, wide-field ODMR detection can provide thermometric performance that are comparable to those provided by confocal detection. Furthermore, we technically estimate multi-point ODMR methods can be integrated into wide-field detection with realistic camera acquisition parameters. We discuss the possibility of realizing large-area spin-based thermometry operable in real-time, which should be an important technological milestone of quantum sensor applications in biology.

## Results and discussion

### Comparison between the detected photon counts in the cases of wide-field and confocal microscopy under the same setup

**Figure 1:**
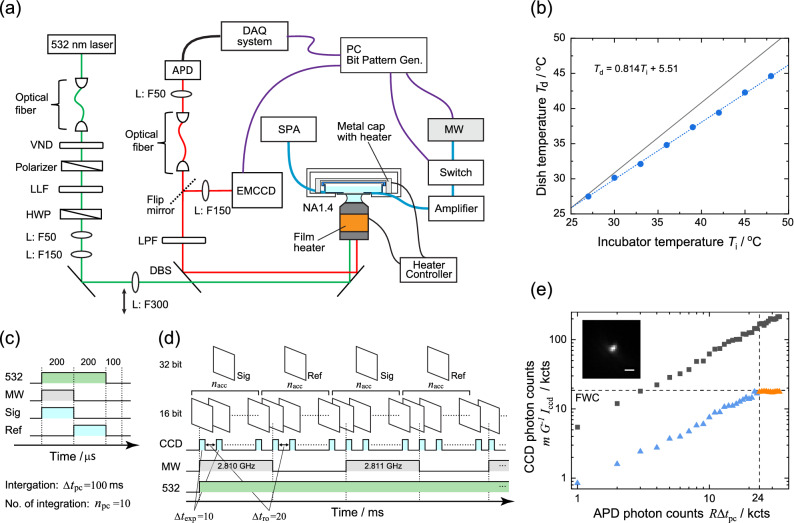
(**a**) Schematic of the ODMR setup including the optical microscope and microwave spin resonance systems. VND: variable neutral density filter LLF: laser-line filter. HWP: half-wave plate. L: lens. DBS: dichroic beam splitter LPF: long-pass filter. EMCCD: electron multiplying charge-coupled device camera APD: avalanche photodiode SPA: Spectrum analyzer. MW: microwave source. DAQ: data-acquisition board. (**b**) Calibration of the dish temperature ($T_\text{d}$) with the incubator heat source ($T_\text{i}$). $T_\text{S} = 5.51 + 0.814 T_\text{i}$ (°C) is obtained. The dotted line represents the linear fit. The solid line indicates a slope of one. Pulse control sequences for (**c**) confocal detection and (**d**) wide-field ODMR detection. Sig: signal. Ref: reference. $\Delta t_\text{ro}$: readout time, 20 ms. $n_\text{acc}$: number of image accumulations, 50 or 100. $\Delta t_\text{exp}$: exposure time of the camera, 10 ms. The frequency sweep in the wide-field ODMR started at 2.810 GHz with a step frequency of 1 MHz. (**e**) The relation between the detected photon counts, under the exposure time of $\Delta t_\text{exp} = 10 \ \text{ms}$, obtained using the camera ($m G^{-1} I_\text{ccd}$) and the APD photon count for an integration time of $\Delta t_\text{pc} = 10$ ms ($R \Delta t_\text{pc}$). Blue and orange triangles indicate single maximal pixel values, whereas gray squares indicate the sum over $6 \times 6$ pixels. At $R \Delta t_\text{pc} = 24$ kcts, $m G^{-1} I_\text{ccd}$ reaches the FWC, thereby exhibiting saturation. (Inset) wide-field fluorescence image of the target ND. Scale bar: 1 $\upmu \text{m}$.

To compare the ODMR spectra of the confocal and wide-field detections, we used a home-built microscope that can perform ODMR measurements in both detection modes (Fig. 1a). The microscope has a home-built stage-top incubator that can store antenna-integrated culture dishes (see “Methods” and Fig. 1b for the temperature profile). In the confocal ODMR detection, the avalanche photodiode (APD) was gated for the ON and OFF states of microwave irradiation with a gate width of 200 $\upmu \text{s}$, which is common to both gates, followed by an initialization time of 100 $\upmu \text{s}$, which gave $I_\text{PL}^\text{ON}$ and $I_\text{PL}^\text{OFF}$ with a frequency of 2 kHz^43^ (Fig. 1c). At each microwave frequency during the frequency sweep, the ND fluorescence was photon-counted for an integration time of $\Delta t_\text{pc} = 100$ ms for a single frequency. The frequency sweep was repeated $n_\text{pc}$ times to obtain a high signal-to-noise ratio (SNR) in the CW-ODMR spectra. For wide-field ODMR observation, we fed the electron multiplying charge-coupled device (EMCCD) camera with trigger pulses from the bit pattern generator to slave its operation (Fig. 1d). The camera acquired a 16-bit image with an exposure time of $\Delta t_\text{exp} = 10$ ms, followed by a readout time of $\Delta t_\text{ro} = 20$ ms. This was repeated $n_\text{acc}$ times for accumulation. The electron multiplication gain (*G*) was set to 10. The obtained 16-bit $n_\text{acc}$ images were summed up into a single 32-bit image to obtain an average (signal image). The microwave was then turned off to acquire the reference image, which was followed by a change in the microwave frequency. The microwave frequency was scanned with a frequency step of $\Delta f_\text{step} =$ 1 MHz in the range of $f_\text{start}$ to $f_\text{end}$. Therefore, we finally obtained a large data set of 32-bit images that contained $2N = 2 (\Delta f_\text{step})^{-1} |f_\text{start} - f_\text{end}|$.

The SNR of the ODMR spectra is dependent on the total photon count measured by the detectors, that is, the EMCCD camera for the wide-field and APD for the confocal detection, and we determined the measurement parameters of wide-field detection that provide a comparable SNR in the confocal method. In wide-field detection, the full-well capacity (FWC) and digital resolution of the analog-to-digital converter (ADC) of the camera strongly limit the measurement strategy. The FWC of our EMCDD is 185,000 e$^-$ for a single pixel, and the ADC resolution is 16-bit expressing the stored electrons in the range of 0–65,535 counts (cts). Taking into account the electron multiplication factor and the near-unity quantum efficiency in the photoelectron conversion process (strictly 90%, based on the manufacturer’s product specification sheet), the camera gives a pixel value of one for $m G^{-1}$ photons per image (*m* is the photon–pixel conversion factor of the camera, given by $185{,}000/65{,}535 = 2.823$). To quantitatively correlate the pixel value with the APD photon count, we measured the fluorescence intensity (photon counts) of single NDs using the EMCCD and APD while the NDs were excited via epi-illumination. The former corresponds to wide-field detection, whereas the latter (the APD detection under the epi-illumination) is called semi-confocal detection in this paper. Figure 1e shows a relation between the photon counts registered in the camera ($m G^{-1} I_\text{ccd}$) and the APD photon count ($R \Delta t_\text{pc}$), where $I_\text{ccd}$ indicates either the maximum pixel value in a region of interest (ROI) encompassing the fluorescence of a single ND or the sum of the pixel values over the ROI for an exposure time of $\Delta t_\text{exp} =10$ ms. Further, *R* and $\Delta t_\text{pc}$ indicate the APD photon count rate (cps: counts per second) and photon counting time (10 ms), respectively. As shown, $m G^{-1} I_\text{ccd}$ linearly increases for both the maximal single pixel value and the sum over the ROI, whereas it saturates beyond 24 kcts at the FWC for a single pixel. From this data, we determined the optical throughput of the pinhole to be $\sim$16% by considering a photon count in the camera and the corresponding APD count (e.g. 127 and 20 kcts, respectively). Thus, wide-field ODMR detection should provide an SNR similar to that of confocal detection when integrating the EMCCD photon counts up to the APD photon counts multiplied by a factor of $6.25 = 0.16^{-1}$. Note that one may expect to use a higher number of photons beyond the FWC because there is no noticeable saturation effect in the summed pixel values. However, ODMR experiments with a high photon flux beyond the FWC cause a frequency shift artifact, as described in the following section. Pixel-saturated NDs cannot be used for thermometry.

### Comparison between wide-field and confocal ODMR detection under the same setup

The aforenoted photon count relation allows us to relate the temperature sensitivities of confocal and wide-field detection. The temperature sensitivity is given by^35,49^

1 $$\begin{aligned} \eta _\text{T} = \frac{\Delta \omega }{|dD/dT|} \frac{1}{C \sqrt{N_X}}, \end{aligned}$$

where *C*, $\Delta \omega$, $N_X$, and *dD*/*dT* indicate the ODMR contrast, ODMR linewidth, total detected photon count corresponding to either of the detectors ($N_\text{APD}$ or $N_\text{CCD}$), and the temperature dependence of the ODMR frequency, respectively. In confocal detection, the total photon count detected by the APD for the ODMR spectral measurements is given by

2 $$\begin{aligned} N_\text{APD} = n_\text{pc} R \Delta t_\text{pc}, \end{aligned}$$

because single measurements ($R \Delta t_\text{pc}$) for individual frequencies are accumulated $n_\text{pc}$ times. In the following ODMR measurements, we used $R = 2$ Mcps, $\Delta t_\text{pc} = 100$ ms, and $n_\text{pc} =10$, giving $N_\text{APD} = 2$ Mcts. By contrast, the total detected photon count in EMCCD is given by

3 $$\begin{aligned} N_\text{CCD} = n_\text{acc} \int _\text{ROI} m G^{-1} I_\text{ccd}(x, y, \Delta t_\text{exp}) dxdy, \end{aligned}$$

and we used the parameters $\Delta t_\text{exp} = 10$ ms and $n_\text{acc} = 100$, giving $N_\text{CCD} = 12.7$ Mcts. With these measurement parameters, we performed ODMR measurements for both detection methods for the same NDs.

**Figure 2:**
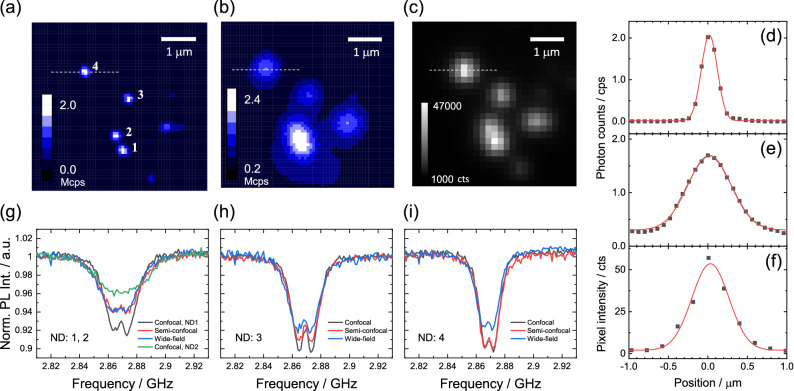
Fluorescence images of the NDs measured using (**a**) confocal scanning, (**b**) semi-confocal scanning, and (**c**) wide-field methods. Cross-sectional plots along the dashed lines in the fluorescence images with the Gaussian fitting curves (red) for (**d**) confocal, (**e**) semi-confocal, and (**f**) wide-field methods. The FWHM values are 0.24, 0.64, and 0.55 $\upmu \text{m}$, respectively. ODMR spectra measured using the three methods for the designated NDs in Fig. 2, as (**g**) **1**–**2**, (**h**) **3**, and (**i**) **4**. Note that NDs **1** and **2** cannot be spatially resolved using semi-confocal and wide-field detection methods. For the measurements, $n_\text{acc} = 100$, $\Delta t_\text{exp} = 10$ ms, $n_\text{pc} = 10$, and $\Delta t_\text{pc} = 100$ ms were used.

Figure 2a–c show the fluorescence images of the NDs in confocal scanning (point excitation and pinhole detection), semi-confocal scanning (wide-field excitation and pinhole detection), and wide-field imaging (wide-field excitation and camera detection), respectively. As can be inferred from the cross-sections shown in Fig. 2d–f, confocal scanning provides the best spatial resolution, while semi-confocal scanning and wide-field imaging provide relatively poor resolutions (similar to each other). Figure 2g–i show the ODMR spectra measured using the three methods for the NDs designated as **1**–**4**, (**1** and **2** are not resolvable in Fig. 2b,c), respectively. In all of the cases, confocal detection provided a relatively better ODMR depth by a factor of $\sim 1.3$, compared with those measured in wide-field detection. The difference in the ODMR depth between the detection methods arises from the background contribution of the fluorescence to the ODMR detection. The background fluorescence is not microwave active, and only acts as the offset of the ODMR spectrum as discussed previously in the context of nanophotonic-device integration of NDs^50^, for example. The semi-confocal detection provided intermediate results compared to the other two methods; the ODMR depth depends on the object shape and size, reflecting how much background fluorescence is included. It provides similar depths to the wide-field results for NDs **1** and **2** because of the large spot size (Fig. 2g), while it improves compared to the wide-field (and reaches the ODMR depth of the confocal results) for NDs **3** and **4** owing to their isolated spots (Fig. 2h,i). The temperature sensitivity can then be calculated from the ODMR linewidth, contrast, and the detected photon counts ($N_\text{APD} = 2$ Mcts and $N_\text{CCD} = 12.7$ Mcts via Eqs. 2 and 3) with a representative temperature dependence of zero-field splitting ($dD/dT = -74 \ \text{kHz} / K$^31,32^) via Eq. (1). In the case of a ND**3**, for which the contrast change among the three methods is the most noticeable, the sensitivity is 2.1, 2.9, and 1.2 K/$\sqrt{\text{Hz}}$ for the confocal, semi-confocal, and wide-field cases, respectively. The large number of photon counts in the wide-field detection provides relatively higher sensitivity than those in the (semi-) confocal detection. Note that it is known that the EMCCD adds additional noise factor of $\sqrt{2}$ originated from the fluctuations of the multiplication gain, slightly degrading the sensitivity to 1.7 ($1.2 \sqrt{2}$) in a more strict way^51^. It should be mentioned that the difference in the spatial resolution between the confocal and wide-field cases is large compared to the theoretical difference; theoretically, the spatial resolution is approximately 30% smaller in the confocal case. While the reason for this discrepancy is not clear, it may be caused by the positional uncertainty in the *z*-axis in wide-field detection. By improving the focusing capability in the wide-field setup, the ODMR contrast degradation may be alleviated.

### The effect of ROI and pixel saturation to ODMR under the positional drift of the NDs

**Figure 3:**
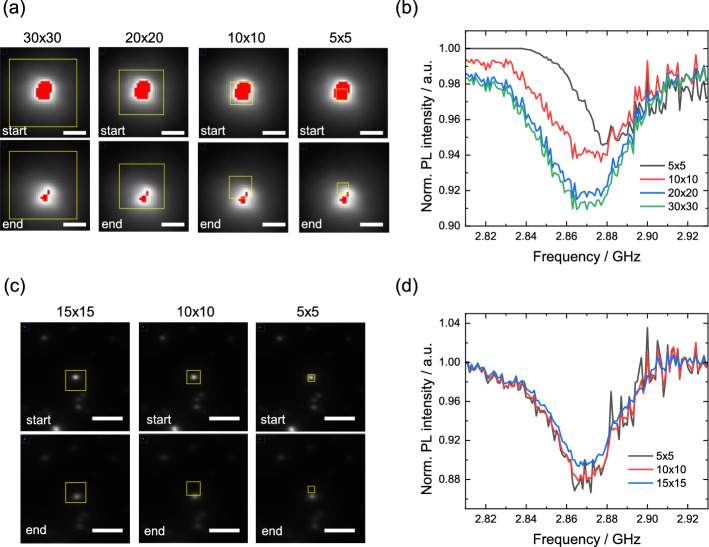
(**a**) Fluorescence images of a single ND with fluorescence saturation at $f_\text{start} = 2.81$ GHz and $f_\text{end} = 2.93$ GHz in the frequency sweep with different ROIs ($30 \times 30$, $20 \times 20$, $10 \times 10$, and $5 \times 5$ pixels). Scale bar: 2 $\upmu \text{m}$. (**b**) Corresponding ODMR spectra of the four types of binning regions. (**c**) Fluorescence images of a single ND without fluorescence saturation at the start and end of the frequency sweep with different ROIs ($15 \times 15$, $10 \times 10$, and $5 \times 5$ pixels). Scale bar: 5 $\upmu \text{m}$. (**d**) Corresponding ODMR spectra of the three types of ROIs. For all the experiments, $n_\text{acc} = 50$ and $\Delta t_\text{exp} = 10$ ms were used.

We subsequently analyzed the influence of the selection of the ROI and the presence of pixel saturation in the camera on the results of ODMR. This is because the ODMR data obtained for pixel-saturated NDs may be used for thermometry, as Fig. 1e does not show a significant effect of pixel saturation on the total detected photon counts, which may be useful, particularly under the positional drift of the NDs because the drift of NDs frequently occurs in biological samples owing to the dynamic change in structures and locomotion^25,44^. Figure 3a,b show images of a single ND at $f_\text{start} = 2.81$ GHz and $f_\text{end} = 2.93$ GHz of the frequency sweep in the ODMR measurement for different sizes of ROIs and the corresponding ODMR spectra, respectively. The ND drifted in the *xyz* directions during a frequency sweep of approximately 6 min. We set four types of gradually decreasing ROIs: $30 \times 30$, $20 \times 20$, $10 \times 10$, and $5 \times 5$ pixels. As the ROIs become smaller, the drifted ND moves out. Accordingly, the ODMR spectra exhibit a decrease in the contrast and shift of the center frequency, which are particularly prominent below $10 \times 10$ pixels. Conversely, the spectral shape of the ODMR is not affected by positional drift when there is no saturation in the ROIs. Figure 3c,d show the fluorescence images of a single ND without saturation and the corresponding ODMR spectra, respectively. As the ROI decreases, the ODMR spectrum associates substantial noise, particularly in the higher frequency side, as the fluorescence spot cannot stay inside the ROIs because of the lateral positional drift.

**Figure 4:**
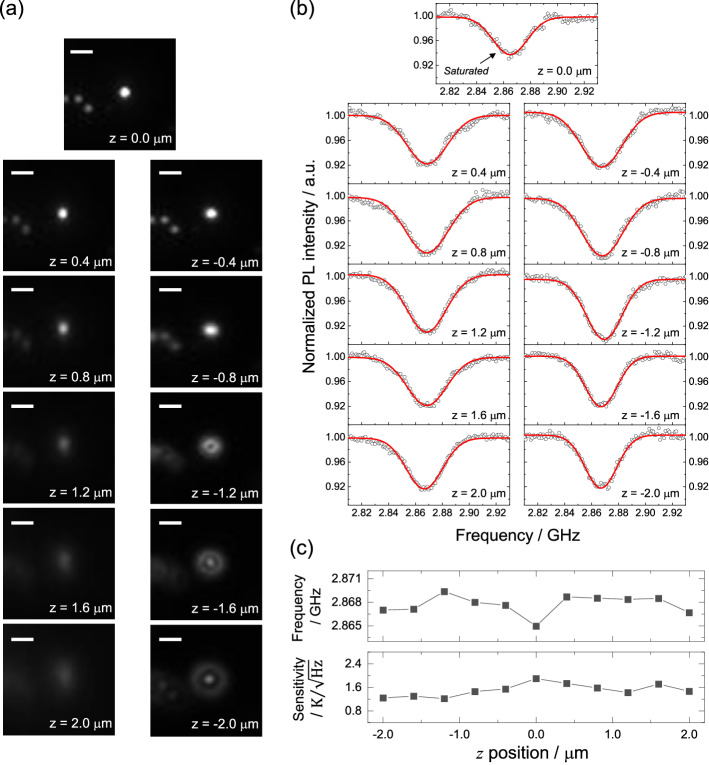
(**a**) Fluorescence images of a single ND with *z*-positional shift of $\pm \ 2 \ \upmu \text{m}$ with respect to the exact focus ($z = 0 \ \upmu \text{m}$) and (**b**) the corresponding ODMR spectra. Scale bar: 2 $\upmu \text{m}$. (**c**) Variation of the central frequency of the ODMR spectra (top) and the estimated temperature sensitivity. $n_\text{acc} = 50$ and $\Delta t_\text{exp} = 10$ ms were used for all the measurements. Note that the fluorescence spot was saturated at $z = 0$ to obtain a sufficient SNR at the most defocused place of $z = \pm 2 \ \upmu \text{m}$.

Next, the effect of the *z*-positional variation of the NDs is characterized. We consider this factor because (1) the mechanical distortion of the microscope system and intra-cellular transportation move the NDs in the *z* axis, and (2) there are a number of blurred ND spots (positional variation in the *z* axis) in a single focal plane owing to the cellular height. Figure 4a,b show a set of fluorescence images of a single ND for the *z*-positional variation of $\pm \ 2 \ \upmu \text{m}$ and the corresponding ODMR spectra, respectively. As the *z*-position is shifted, the ND is defocused, exhibiting the 3D shape of the point spread function. Figure 4c shows the center frequency of the ODMR spectrum at each *z*-position determined by Gaussian fitting and the corresponding temperature sensitivity estimated based on the spectral shape and the photon count rate. The center frequency exhibits a fluctuation of 370 kHz (s.e.), except at $z = 0$. The net fluctuation is consistent with that obtained previously for the confocal detection^52^, whereas the deviation at $z = 0 \ \upmu \text{m}$ is an artifact from pixel saturation. The sensitivity data are steady and provide a mean of 1.5 K/$\sqrt{\text{Hz}}$ for this particular ND.

The present results regarding the dependence of the wide-field ODMR on the positional variation of the NDs can be summarized as: (1) The measurement noise (or temperature precision) is dependent on the ND fluorescence flux summed up in the ROI. (2) The blurred fluorescence spots of individual NDs at a single focal plane do not create artifacts of ODMR shift as long as the ROI is properly set. (3) The pixel saturation of the camera requires particularly careful treatment, as it may cause measurement artifacts. Increasing the fluorescence intensity and number of measurable NDs in a single image is an important factor in achieving high SNR, but it inevitably saturates the fluorescence of the brighter NDs. This is because the excitation power is usually adjusted such that the majority of the NDs gain 40,000–50,000 pixel values. There should be a tradeoff between the efficient optical excitation power and the number of available NDs depending on the real experiments on biological samples. Parameter optimization, such as the adjustment of optical magnification and exposure time, is not always possible in a dynamic biological environment; this necessitates the present characterization of pixel saturation such that it can be effectively addressed. Note also that the splitting of ODMR dip varies by particles because of the differences in transverse component of zero-field splitting parameters and effective microwave power relative to the saturated intensity. These spectral variations can affect the curve-fitting results, leading to a variation in the sensitivity and precision.

### Three dimensional distribution of NDs in HeLa cells

**Figure 5:**
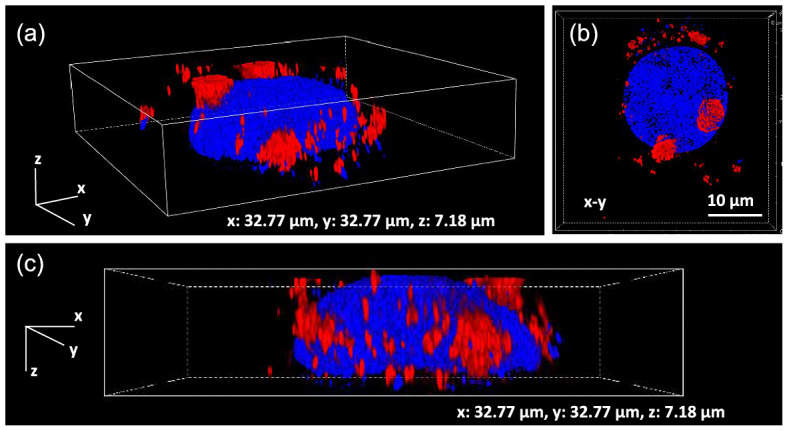
Super-resolution fluorescence image of ND-labeled HeLa cells fixed on coverslip. (**a**) Three-dimensional fluorescence images of the nucleus (blue) and NDs (red). (**b**,**c**) Corresponding two-dimensional images of *xy* and *xz*.

Before working on cells, we quantify the *z*-positional distribution of NDs in HeLa cells (and their spatial distribution in the *xy* plane). We performed super-resolution imaging of ND-labeled HeLa cells that were fixed on a coverslip, as shown in Fig. 5. Typically, HeLa cells have a two-dimensional size of $40 \times 40 \ \upmu \text{m}^2$; furthermore, they have a height of $7 \ \upmu \text{m}$ near the nucleus. The NDs around the nucleus were uniformly distributed. It is important to note that the distribution of NDs in cells significantly depends on cell type. For example, adipose-tissue-derived stem cells have significantly flat structures ($30 \times 30 \ \upmu \text{m}^2$ in the *xy* plane and $2 \ \upmu \text{m}$ in *z*)^43^, and more NDs can be focused in the wide-field fluorescence image in contrast to HeLa cells.

Although there are approximately 200 NDs in the cells, not all NDs can be used for thermometry because (1) some NDs (approximately 20–30%) show broad spectral lines that cannot provide high sensitivity (particularly, peak-shift detection is difficult) and (2) practically, only 10–20% of the NDs can be focused in the tolerable focusing range in the *z*-direction because of the substantial height of cells in the *z* axis compared to the focal depth. In the subsequent experiments involving wide-field ODMR measurements in living ND-labeled HeLa cells, 10–30 NDs were predominantly available for measurements. Note that the number of NDs inside cells can be increased by employing a higher ND concentration. The selection of the ND concentration is dependent on the purpose. In our experiment, we used a relatively low concentration to focus on individual NDs so that the principles of the system, with regard to the wide-field ODMR detection relative to the confocal one, could be investigated.

### Wide-field ODMR and intracellular temperature measurements in HeLa cells

**Figure 6:**
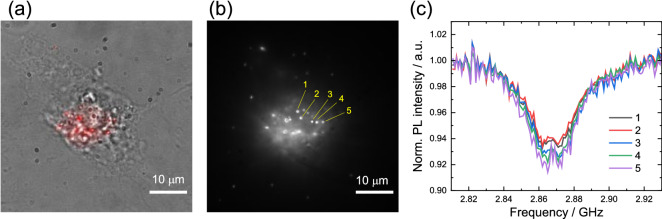
Microscope images of living ND-labeled HeLa cells; (**a**) merged (bright field and red fluorescence) and (**b**) red fluorescence only. (**c**) ODMR spectra of NDs **1**–**5**. $n_\text{acc} = 50$ and $\Delta t_\text{exp} = 10$ ms were used for all the measurements.

Having understood the system principles of wide-field detection in detail and its connection to confocal detection, we apply it to multiple NDs in living HeLa cells. Figure 6a,b show merged (bright-field and red) and red fluorescence images of the ND-labeled HeLa cells, respectively. In these images, we observed $\sim 20$ clear spots of NDs, and a total of $\sim 50$ NDs were discernible at a single focal plane. We then performed wide-field ODMR measurements and obtained the ODMR spectrum of multiple NDs by setting the ROIs as described above. Figure 6c shows the representative ODMR spectra of the NDs designated as **1**–**5** in Fig. 6b. Interestingly, the ODMR spectra of the fluorescence spots are similar to those of each other, and the particle-dependent ODMR inhomogeneity appears to be less significant than that of the NDs on coverslip. This is most probably because multiple NDs are encapsulated in single endosomes^53^. Indeed, the fluorescence spots of the NDs are sometimes merged or overlapped during the measurements because of the intra-cellular transportation and merger of endosomes. NDs **1** and **2** are insignificantly fluorescence-saturated and doughnut shaped owing to the electron overflow in the pixel arrays. This saturation effect appears as the relatively small ODMR contrasts of NDs **1** and **2**, compared to those of NDs **3**–**5**. Accordingly, the estimated sensitivities for NDs **1**, **2** are 2.1 and 2.2 K/$\sqrt{\text{Hz}}$, and these are relatively worse than those for NDs **3**–**5**, which show 1.9, 1.8, and 1.7 K/$\sqrt{\text{Hz}}$, respectively. These sensitivity values are in agreement with the previous reports^23,54^ and relatively worse than those on the coverslip in this study because (1) the background fluorescence reduces the ODMR contrast and (2) the ODMR spectra are broad in cells.

**Figure 7:**
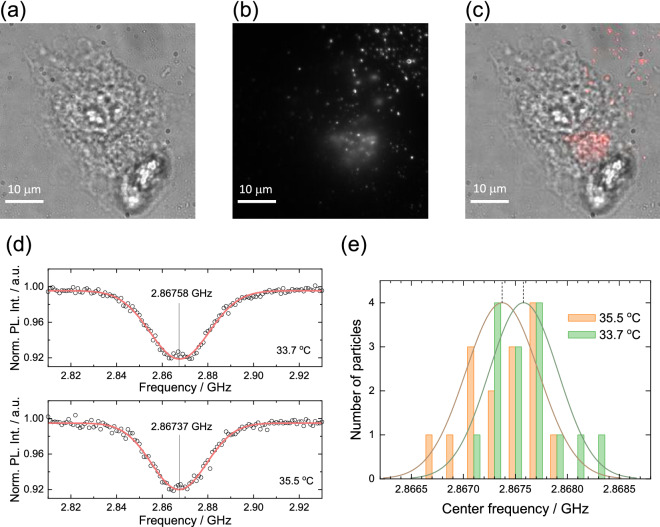
Microscope images of the ND-labeled HeLa cells; (**a**) bright field (**b**) red fluorescence, and (**c**) merged. Scale bar: 10 $\upmu \text{m}$. (**d**) ODMR spectra averaged over 15 NDs in the cell at $T_\text{d} =$ 35.5 (bottom) and 33.7°C (top). (**e**) Histograms of the center frequency of 15 NDs at the two temperatures with normal distribution fitting. $n_\text{acc} = 50$ and $\Delta t_\text{exp} = 10$ ms were used for all the measurements.

We then performed intracellular temperature sensing of living HeLa cells in the present wide-field detection. Figure 7a–c show another set of bright-field, red-fluorescence, and merged images of the ND-labelled HeLa cells at 35.5°C, respectively. The dish temperature, $T_\text{d}$, is then varied from 35.5 to 33.7°C, and the ODMR spectra of the NDs were measured using wide-field detection at each temperature. Figure 7d shows the ODMR spectra averaged over 15 ND fluorescence spots inside the cell at these temperatures. The center frequency of the ODMR spectrum is shifted by 210 kHz when the dish temperature is varied by $-1.8^{\circ}{\rm C}$. The fitting errors of the Gaussian function to the ODMR spectra at $T_\text{d} =$ 35.5 and 33.7°C are 135 and 165 kHz, respectively. To further confirm the ODMR shift due to the temperature change, we statistically analyzed the temperatures of individual NDs. Figure 7e shows the histograms of the center frequency of 15 NDs at the two temperatures, where the center frequency is determined by Gaussian fitting. The ND temperature indication shows a normal distribution and provides center frequencies of 2.86737 and 2.86758 GHz at $T_\text{d} =$ 35.5 and 33.7°C, respectively. The standard errors of the center frequencies were 86 and 91 kHz, respectively. Assuming $dD/dT = -74$ kHz/K, the temperature change sensed by multiple NDs can be calculated as $- 2.8$ K with an uncertainty of $\sim 1.2$ K.

The present ND thermometry detected the temperature change; however, the accuracy and precision are limited because of the difficulties in the CW-ODMR thermometry method as indicated in the confocal detection^43^. The ODMR linewidth (10–30 $\text {MHz}$) is approximately two-order magnitude larger than the temperature dependent shift ($\sim 100\; \text {kHz}$) that limits the precision of the ODMR frequency determination, i.e., fitting error. The ODMR split structure derived from the NV ensemble further degrades the precision^23^. Without enhancing the accuracy and precision, it is challenging to state cellular thermal responses. However, it may be possible to address this limitation by implementing multi-point ODMR methods^31,48^. Recently, we showed that the four-point ODMR thermometry techniques used for confocal detection can provide a temperature precision of 0.22 K in worms (1.4 $\text{K}/\sqrt{\text{Hz}}$)^44^, whereas it only yields a precision of approximately 3 K (2.2 $\text{K}/\sqrt{\text{Hz}}$) in the same setup using the CW-ODMR method^43^. We discuss the possibility of implementing the multi-point ODMR methods in the wide-field detection in the next section. Note that tracking the same NDs between the temperature measurements may improve the temperature precision. Note also that the NDs act as relative thermometers that measure the difference between two temperatures. The possible heat generation by microwaves is particularly eliminated if the microwave absorption of water does not change substantially. In addition, we showed that the buffer temperature in the dish was stable within 0.2K at the present microwave power using the thermistor (see “Methods”); the heating effect of microwave absorption was not affected in the present experiments.

### Prospective to extend to real-time measurements for thermal live-imaging of cells

The present study has clarified that wide-field ODMR detection can provide comparable results with confocal detection, as long as the background fluorescence is low and the pixel saturation is properly treated. The selection of ROIs and the fluorescence brightness of the NDs that lead to pixel saturation are critical when implementing wide-field ODMR detection in biological thermometry. This technical point is camera dependent, where higher bit depths and larger FWCs are required for the camera, as reported for magnetometry applications^55^. For example, single-photon counting cameras^56^ or neural imaging cameras^57^ may be useful for such applications. On the other hand, most of scientific CMOS cameras have relatively small FWC and has a challenge to enhance the sensitivity as discussed in the context of magnetometry^55^, while they usually have smaller noise; therefore the choice of camera is truly application dependent. While wide-field detection is robust against the *z*-positional variation of the NDs, coarse positional tracking is necessary to obtain full-3D volumetric temperature measurements of entire cells. Combining such *z*-positional information with phase imaging techniques^58^ can provide more precise information about cellular structures, which could aid subcellular temperature mapping^9,10^. Additionally, the reduction of the measurement times of the ODMR spectra is important. For example, the fast-timing image detection mode of the camera may speed up this process. Integrating wide-field detection with multi-point ODMR methods seems necessary to improve the temperature precision, as confocal detection has recently demonstrated biological thermometry in living nematode worms upon integration with multi-point ODMR^44,45^. From the present results, we can calculate realistic acquisition parameters for wide-field detection. Previously, we performed the 4-point ODMR measurements for confocal detection using the same experimental setup, with parameters of $R = 2$ Mcps, $\Delta t_\text{pc} = 100 \ \upmu \text{s}$, and $n_\text{pc} =2380$^44,48^. Considering the degradation of ODMR contrast by a factor of $\sim 1.3$, the corresponding sensitivity in the wide-field detection may be obtained with $\Delta t_\text{exp} = 10$ ms and $n_\text{acc} \sim 10$ for each of the four frequencies. Note that there are other approaches to speed up the wide-field ODMR detection, which may be a choice for the real-time measurements^59,60^.

In addition to the development of these measurement techniques, the spatial distribution of NDs strongly affects the measurement strategy. In the present study, the NDs were freely distributed in the cells. In such cases, one can obtain the temperature information of whole cells or certain regions of cells, as reported previously^26,43–45^. One such example is the recent demonstration of spin-based thermometry of subcellular temperature gradients in embryos^45^, where the spatial distribution of temperature inside embryos is measured using freely distributed NDs while controlling the local temperature. With site-specific ND labeling techniques, one may control this ND spatial distribution more effectively. There are various types of biomolecular conjugation of NDs that enable site-specific temperature measurements of various organelles, such as mitochondria^61^, cellular membrane^62^, and lysosomes^63^. Intra-cellular spin-based thermometry thus requires the development of both measurement techniques and ND labeling techniques for target biological phenomena.

In conclusion, we have analyzed wide-field spin-based ND thermometry in detail for application in intra-cellular temperature measurements. The ODMR spectra obtained using confocal and wide-field detection were compared for the same NDs, and we found that the ODMR was deeper in the confocal detection than in the wide-field detection. However, the associated degradation of measurement sensitivity is compensated by the high photon flux in the wide-field detection, resulting in relatively better sensitivity in the present setup. The effect of pixel saturation and ND positional drift on the wide-field ODMR detection was studied to examine measurement artifacts and sensitivity degradation. From this system characterization, we adjusted the measurement parameters and performed ODMR measurements of multiple NDs in living HeLa cells. Although the external temperature change can be detected with the comparable precision as that measured in the confocal detection, further improvements in temperature precision are necessary. We calculated realistic measurement parameters for the integration of multi-point ODMR methods with wide-field detection and discussed the further development of spin-based ND thermometry for real-time large-area thermometry for microscopic biological applications.

## Methods

### Materials

The NDs (NDNV100nmHi10ml) were purchased from Adámas Nanotechnologies (aleigh, VA, USA), and they contained approximately 500 NV centers per particle. E-MEM with l-glutamine, phenol red, sodium pyruvate, non-essential amino acids, sodium bicarbonate (1500 mg/L), Dulbecco’s phosphate-buffered saline (D-PBS(-)), and 4% paraformaldehyde phosphate buffer solution were purchased from Fujifilm Wako Pure Chemical Corporation (Tokyo, Japan). D-MEM and fetal bovine serum (FBS) were purchased from Thermo Fisher Scientific (Waltham, MA, USA). Collagenase type I was purchased from Koken Co. (Tokyo, Japan). A cell counting plate was purchased from Fukae Kasei Co. (Hyogo, Japan). Acti-stain 488 phalloidin was purchased from Cytoskeleton (Denver, CO, USA). Hoechst 33342 was purchased from Thermo Fisher Scientific (Tokyo, Japan). Fluoromount was purchased from Diagnostic BioSystems (Pleasanton, CA, USA).

### Cell preparation and ND labeling for super-resolution microscopy

HeLa cells were incubated with the NDs (250 $\upmu \text{g}$/mL) on glass-based dishes in a transduction medium (D-MEM containing 10% FBS and 100 U/mL penicillin/streptomycin) at 37°C. After a 24-h incubation, the cells were washed two times using PBS. The cells were fixed with 4.0% paraformaldehyde for 30 min. The nuclei of the ND-HeLa cells were then stained with Hoechst 33342 solution for one h. After these staining treatments, the cells were washed twice using PBS. Finally, the cells were soaked in PBS. The stained ND-HeLa cells were observed to obtain the three-dimensional distribution of NDs using a super-resolution structured illumination microscope (N-SIM; Nikon). The excitation and emission wavelengths were 325/400–450 nm and 550/600–700 nm for the nucleus and NDs, respectively.

### Cell preparation and ND labeling for ODMR measurements

HeLa cells were incubated with the NDs (10 $\upmu \text{g}$/mL) in home-built antenna-integrated glass-based dishes in a transduction medium (E-MEM containing 10% FBS and 100 U/mL penicillin/streptomycin) at 37°C. The dishes were collagen-coated before use. After a 24-h incubation, the cells were washed three times using PBS solution and filled with the transduction medium without phenol red. The phenol red was removed to avoid heat generation upon the absorption of the green laser light.

### Microscope and microwave setup of spin-based thermometry

A continuous-wave 532-nm laser was used for optical excitation. An oil-immersion microscope objective with a numerical aperture of 1.4 was used for the excitation and fluorescence collection. The NV fluorescence was filtered with a dichroic beam splitter, and a long-pass filter was used to remove residual green laser scattering. For confocal detection, the fluorescence was coupled to an optical fiber that acted as a pinhole (1550HP, Thorlabs). The fiber-coupled fluorescence was detected using APD (SPCM AQRH-14, Excelitas). For wide-field ODMR detection, the excitation laser was focused on the back focal plane of the objective to illuminate the entire area of the field of view, and the fluorescence was imaged with an EMCCD camera (Evolve Delta, Photometrics) via a *f*150-mm achromatic lens. The electron multiplication gain was set to 10. In both detection methods, the excitation intensity was adjusted to $\sim 10 \ \text {W} / \text{cm}^{2}$. The sample was mounted on an *xyz*-piezo stage (Piezosystemjena, Tritor-100SG) to enable fine translation and positional scanning. Note that an external magnetic field was not applied in this study. Microwaves were generated from a microwave source (SMB100A, Rohde & Schwarz) and amplified by a maximum factor of 45 dB (ZHL-16W-43+, Mini-circuit). The microwaves were fed to the linear antenna (25-$\upmu \text{m}$ copper wire) of the home-made cell culture dishes. The typical microwave excitation power was estimated to be 10–50 mW (10–17 dBm) at the linear antenna by considering the source output, amplifier gain, and the experimentally determined $S_{21}$ of the antenna system, which provides a microwave magnetic field of more than 2–5 gauss in 20 $\upmu \text{m}$ from the antenna. APD detection was gated for ON and OFF states of microwave irradiation using a radiofrequency switch (ZYSWA-2-50DRS, Mini-circuit) and bit pattern generator (PBESR-PRO-300, Spincore).

### Image analysis

The obtained $2N = 2 (\Delta f_\text{step})^{-1} |f_\text{start} - f_\text{end}|$ image sets were first sorted according to the respective microwave frequencies, and further according to the two types of *N* image sets for the microwave ON and OFF states. To obtain the ODMR contrast, pixel values of the microwave ON images (signal images) were divided by those of the microwave OFF images (reference images). This image data processing was performed using Fiji/ImageJ software^64^. The obtained ODMR spectra were fitted with Gaussian in a practical manner because there are no simple analytic expressions for the ODMR spectral shape of ensemble NV centers^65^.

### Temperature control of the stage-top incubator

In the home-built stage-top incubator, we heated the antenna-integrated dishes in two directions, that is, from the direction of the oil-immersion objective and metal cap (Fig. 1a). The temperature of the dish ($T_\text{d}$) was varied by controlling the temperature of the heat sources that were set to the same temperature ($T_\text{i}$) using the PID-feedback controller of the foil heater, which wrapped the objective (Thorlabs, HT10K & TC200, temperature precision: ± 0.1K). The immersion oil used was an Olympus Type-F. $T_\text{d}$ was calibrated by inserting a tiny flat Pt100 thermistor (Netsushin, NFR-CF2-0505-30-100S-1-2000PFA-A-4, $5 \times 5 \times 0.2 \ \text{mm}^{3}$) in the water media in the dishes, and varying $T_\text{i}$ while monitoring $T_\text{d}$. The thermistor was read using a high-precision handheld thermometer (WIKA, CTH7000, temperature precision: ± 0.02 K). Consequently, we obtained the following relation, $T_\text{d} = 5.51 + 0.814 T_\text{i}$ (°C), as shown in Fig.  1b. Note that $T_\text{i}$ was monitored directly on top of the foil heater wrapped around the objective and capping metal cover. The temperature stability in this incubator was $\pm 0.25{\rm K}$ over 250 min when measured by the thermistor or $\pm 0.4{\rm K}$ over 140 min when measured by NDs as confirmed in Ref.^48^.
